# In Search of the Executive Cognitive Processes Proposed by Process-Overlap Theory

**DOI:** 10.3390/jintelligence9030043

**Published:** 2021-08-19

**Authors:** Gidon T. Frischkorn, Claudia C. von Bastian

**Affiliations:** 1 Department of Psychology, University of Zurich, Binzmuehlestrasse 14, 8050 Zurich, Switzerland; 2 Department of Psychology, University Sheffield, Sheffield S1 1HD, UK; c.c.vonBastian@sheffield.ac.uk

**Keywords:** intelligence, executive processes, working memory, processing speed, Process-Overlap Theory

## Abstract

Process-Overlap Theory (POT) suggests that measures of cognitive abilities sample from sets of independent cognitive processes. These cognitive processes can be separated into domain-general executive processes, sampled by the majority of cognitive ability measures, and domain-specific processes, sampled only by measures within a certain domain. According to POT, fluid intelligence measures are related because different tests sample similar domain-general executive cognitive processes to some extent. Re-analyzing data from a study by De Simoni and von Bastian (2018), we assessed domain-general variance from executive processing tasks measuring inhibition, shifting, and efficiency of removal from working memory, as well as examined their relation to a domain-general factor extracted from fluid intelligence measures. The results showed that domain-general factors reflecting general processing speed were moderately and negatively correlated with the domain-general fluid intelligence factor (*r* = −.17–−.36). However, domain-general factors isolating variance specific to inhibition, shifting, and removal showed only small and inconsistent correlations with the domain-general fluid intelligence factor (*r* = .02–−.22). These findings suggest that (1) executive processing tasks sample only few domain-general executive processes also sampled by fluid intelligence measures, as well as (2) that domain-general speed of processing contributes more strongly to individual differences in fluid intelligence than do domain-general executive processes.

## 1. Introduction

Process-Overlap Theory (POT; Kovacs and Conway 2016) is a promising account bridging the gap between hierarchical models of intelligence, which typically posit a general intelligence factor *g* on the highest level (Carroll 1993; Horn 1965; Jensen 1998; McGrew 2005; Spearman 1904), and theories that propose several distinct, potentially independent, cognitive processes underlying individual differences in intelligence (Thurstone 1938; Van Der Maas et al. 2006). In its essence, POT is a reformulation of sampling theory of intelligence (Thomson 1916) in its assumption that all intelligence measures sample from a set of cognitive processes that are required for performing well in the respective measures.

Formally, POT builds upon multi-dimensional item-response theory (IRT) to implement individual differences in intelligence as a composite of domain-general and domain-specific cognitive processes. The domain-general processes are thought to be executive processes identified by cognitive experimental research, particularly executive processes in working memory. Domain-specific processes are described as processes required only within a specific content domain (e.g., verbal or visual literacy). Critically, POT assumes that limitations in one domain-specific process can be compensated by other cognitive processes operating within the same domain. In contrast, limitations in one domain-general process cannot be compensated by other domain-general processes. Therefore, performing well in a given task requires a certain level of ability in all the domain-general cognitive processes tapped by that task. Computationally, these relationships are implemented by additive associations between domain-specific cognitive processes, and multiplicative associations between domain-general cognitive processes.

In summary, according to POT, the hierarchical structure of intelligence arises because (1) domain-general executive and domain-specific cognitive processes are separated, (2) domain-general executive processes are, to some extent, required by all cognitive tasks independent of their domain; therefore, (3) domain-general executive processes overlap more strongly with domain-specific cognitive processes than domain-specific processes do with each other. Based on these three central specifications, POT conceptualizes the *g* factor as a formative construct that emerges from sampling overlapping executive processes. This is different from other models that postulate a higher-order *g* factor as a common cause determining individual differences in intelligence.

### 1.1. What Executive Processes Are Proposed by POT?

The main goal of the current article is to identify and investigate the domain-general executive processes that have been predominantly discussed in relation to POT. Here, we focused on fluid intelligence (*Gf*) because POT suggests that *Gf* measures primarily sample executive processes (Kovacs and Conway 2016, 2019), thereby explaining why *Gf* measures tend to show higher loadings on general intelligence than other broad intelligence factors (e.g., crystallized intelligence or perceptual abilities). If POT is correct in assuming that the shared variance of *Gf* measures emerges from the overlap in domain-general executive processes sampled by these *Gf* measures, then measures of domain-general executive processes should uniquely predict individual differences in *Gf*. Hence, a critical test of POT is whether measures of executive processes uniquely and independently explain individual differences in a factor derived from several *Gf* measures. Furthermore, according to POT, these different executive processes should be independent from each other (i.e., uncorrelated) and, thus, should not form a single factor by themselves.^1^

In general, *executive processes* is an umbrella term for a broad set of cognitive functions involving controlled processing (for a review, see Diamond 2013). POT specifically refers to those executive processes that are associated with the central executive in working memory (Baddeley and Hitch 1974; Kovacs and Conway 2016) based on empirical results indicating incremental predictive validity of working memory over short-term memory measures (Conway et al. 2002; Engle et al. 1999; Kane et al. 2004). Complex span and other working memory tasks (e.g., *N*-back tasks) combine storage and processing demands, whereas simple-span tasks used to assess short-term memory involve only storage of information. Hence, these findings suggested that the supervisory attentional control processes managing storage and processing in working memory are critical for explaining individual differences in intelligence.^2^

Conceptually, executive processes and their interpretation as supervisory attentional control processes originated from robust experimental effects in tasks that require inhibiting of incongruent information (Eriksen and Eriksen 1974; Simon et al. 1981; Stroop 1935), switching between tasks (Monsell 2003; Monsell and Driver 2000), or selectively updating information in working memory (Kirchner 1958). These experimental effects suggested that, in addition to basic information processing, cognitive processes exist that regulate and direct the focus of attention when working on cognitive tasks. For example, reaction times (RTs) are typically longer in task conditions that demand ignoring irrelevant incongruent information than RTs in task conditions that demand ignoring irrelevant congruent information. The experimental effect, i.e., the difference in RTs between these two conditions, is considered a manifestation of the attentional control mechanisms implemented in the central executive of working memory (Jurado and Rosselli 2007; Miyake et al. 2000). People exhibiting smaller experimental effects in these tasks are considered to use the required executive processes more efficiently. Consequently, researchers incorporated such tasks to assess individual differences in the ability to regulate attention during information processing.

Most commonly, three executive processes domains are distinguished (Miyake et al. 2000): inhibition, shifting, and updating. *Inhibition* refers to the ability to focus attention on relevant information while ignoring irrelevant information or suppressing prepotent responses, *shifting* is the ability to flexibly switch between different tasks, and *updating* is the ability to efficiently remove outdated information from memory and encode new information into working memory. More recently, researchers further distinguished between three components of updating (Ecker et al. 2010, 2014): retrieval, transformation, and removal of information from working memory. The current study will focus on the latter process, the efficiency of removal, based on previous research showing that this is the executive process that distinguishes updating from basic short-term memory maintenance processes (Ecker et al. 2014).^3^ Although POT does not explicitly refer to these executive processes as the domain-general executive processes that would be sampled by *Gf* measures, other researchers suggested that the ability to disengage from information in cognitive processing tasks plays a critical role in explaining individual differences in *Gf* (Shipstead et al. 2016).^4^

Empirical research on the relationship among different executive processes (Karr et al. 2018; Miyake et al. 2001), such as inhibition, shifting, and updating, suggest that there is both overlap (or unity) and independence (or diversity) between executive processes. Specifically, variance arising from inhibition tasks has been found to fully overlap with variance from performance in shifting and updating tasks, whereas shifting-specific and updating-specific factors additionally capture unique individual differences that are independent from the common executive processing factor (Friedman et al. 2008, 2009, 2016; Fleming et al. 2016; Kramer et al. 2014). These empirical findings only partially support the POT assumption that domain-general executive processes are independent from each other, as all three factors overlap and, thus, form the common executive processing factor. Yet, these measures themselves likely are not process-pure and, therefore, sample several executive processes. In addition, it is important to note that a recent meta-analysis suggests that the majority of factor-analytical studies of executive processes did not have sufficient sample sizes to adequately contrast different factor structures of executive processes (Karr et al. 2018). Hence, robust evidence with respect to the unity or diversity of executive processes is still scarce.

### 1.2. How Are Executive Processes Measured?

A recent attempt to address conceptual and methodological problems in the measurement of executive processes (von Bastian et al. 2020) highlights that considerable diversity exists in what paradigms and scoring methods are used. A particularly heated debate centers around whether to use average performance in tasks or conditions with executive processing demands, or whether variance unique to executive processes should be isolated by contrasting task conditions with high and low executive processing demands. Some researchers argue that difference scores, especially for RTs, are inadequate for investigating individual differences because they assume cognitive processes to be strictly additive and because they often lack reliability (Ackerman and Hambrick 2020; Draheim et al. 2019). One approach to overcome these problems is to develop new tasks that focus exclusively on average accuracy in tasks requiring executive processes (Draheim et al. 2021). Yet, from a theoretical-conceptual perspective, this is not a real alternative to a contrast between experimental conditions, as average accuracy scores will contain variance from both executive and basic cognitive processes. In addition, some studies found acceptable reliabilities of difference scores for assessing executive processes (Rey-Mermet et al. 2018, 2019; Singh et al. 2018; von Bastian and Druey 2017), demonstrating that reliable measurement of isolated executive processes is generally possible.

The most promising alternative to differences scores is to isolate executive processes using cognitive measurement models (Frischkorn and Schubert 2018; von Bastian et al. 2020) that provide a formalized implementation of the cognitive processes assumed to underlie observed behavior. For some executive processing tasks (e.g., the flanker task), several cognitive measurement models have been developed (Huebner et al. 2010; Ulrich et al. 2015; White et al. 2011). Yet, robust estimation of parameters for these models is still difficult (Hübner and Pelzer 2020), and such cognitive measurement models do not exist for all common executive processing tasks.

**Figure 1:**
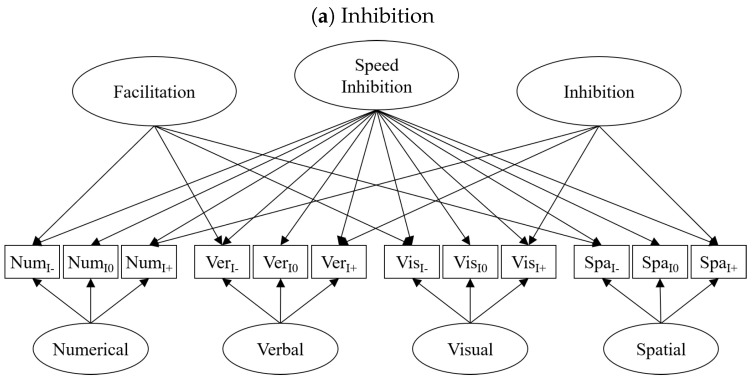

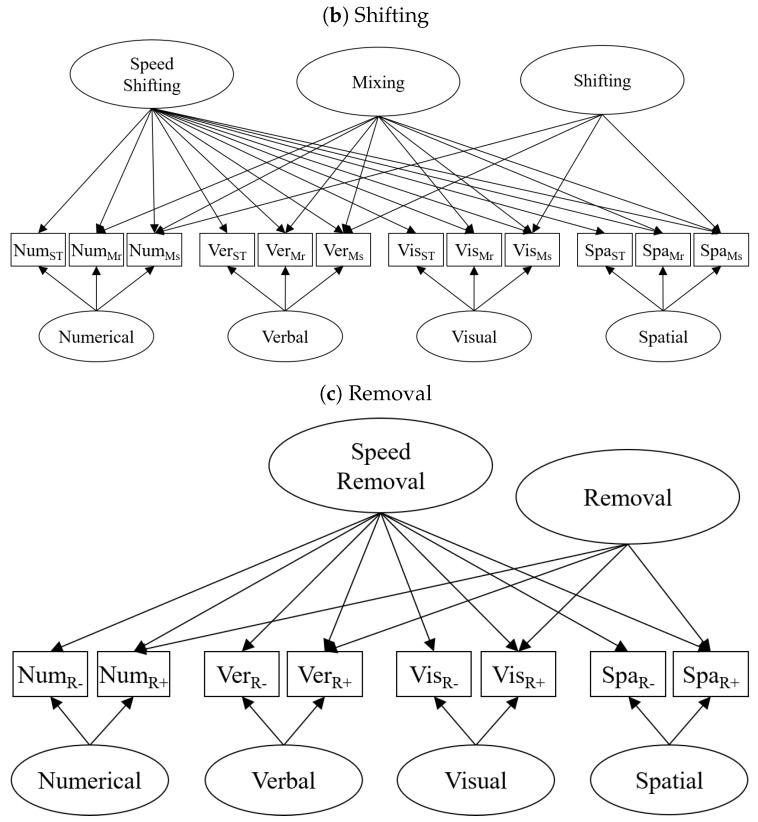
Simplified path diagrams of bi-factor models isolating condition specific individual differences from baseline processing speed in executive processing tasks. In addition, the models include specific factors that capture individual differences unique to a single task or a content domain. For inhibition and removal tasks, the *+* index refers to a condition strongly demanding the respective executive process, whereas an *-* index suggest that it demands it less. For the shifting tasks, single task (ST) performance was separated from performance in mixed blocks (M) for trials repeating the task of the previous trial (r) and trials switching to a different task than in the previous trial (s). Error variances were omitted in the figure to ease readability.

The next best yet readily accessible option for isolating only the reliable portion of condition differences in executive processing tasks is to use elaborate statistical methods, such as structural equation models (SEM; McArdle 2009; McArdle and Woodcock 1997). By isolating only reliable variance proportions from unsystematic error variance (e.g., trial noise or other sources of measurement error), such models are capable of isolating condition-specific individual differences that are, consequently, also perfectly reliable. One option to implement such models is a bi-factor approach (see Figure 1). In bi-factor models, a general factor extracts the variance common to all indicators; in this case, these are the different conditions in the executive processing tasks. In addition, a condition-specific factor isolates the variance unique to one condition and, thus, represents individual differences in executive processes that are more strongly tapped by this condition compared to the baseline condition. Moreover, if there are multiple tasks tapping the same executive processes, task-specific factors capturing individual differences specific to each task or material used in the task can be isolated. Although such models assume that cognitive processes are associated in an additive manner, thereby diverging from POTs assumption of multiplicative association, this is the most suitable way to combine the conceptual foundations of executive processes as condition-differences and the need for reliable measures of individual differences in these processes. In particular, this is because the current study is not interested in testing whether executive processes are associated additively or multiplicatively, but only whether a general fluid intelligence factors is uniquely related to different executive processing factors.

### 1.3. The Present Study

To address the question whether different executive processes are independently related to individual differences in *Gf*, we re-analyzed data from a previous study (De Simoni and von Bastian 2018). Tasks administered in this study included four measures each tapping inhibition, shifting, and removal of information from working memory, and four *Gf* measures. The aim of the re-analysis in the present study was to measure individual differences in domain-general executive processes and to assess how these are related to individual differences in fluid intelligence.

According to POT, the *Gf* factor, which reflects individual differences in domain-general cognitive processes sampled by the *Gf* measures, should be related to domain-general factors from the executive processing tasks. Furthermore, the domain-general factors isolating variance specific to the experimental conditions with high executive-processing demands should correlate particularly strongly with domain-general *Gf*. In addition, if POT is correct in assuming that there is no single cognitive process underlying the *Gf* factor, the different domain-general executive processing factors should independently predict individual differences in *Gf*.

## 2. Methods

### 2.1. Participants

We re-analyzed data from a cognitive training study published by De Simoni and von Bastian (2018). As training may affect interrelations between the cognitive measures, we analyzed only the data from the pretest (N=237). The participants were young adults (68.4% female, ♀162, ♂75) between 18 and 36 years old ($M_{age}=23.86$, $SD_{age}=3.83$), who were reimbursed with 120 CHF (approximately $125), or 10 course credits and 20 CHF (approximately $21), after completing the study. Participants were from heterogeneous, but mainly higher educational, backgrounds (75.1% had a high school diploma, 19.4% had a college degree, 3.0% had a degree from an applied university, about 2% finished and apprenticeship or had a technical high school degree, and 0.4% obtained a doctorate). For further details on the sample, see De Simoni and von Bastian (2018).

### 2.2. Cognitive Measures

In the original study (De Simoni and von Bastian 2018), participants’ cognitive abilities were assessed with 28 computer-based tasks before and after a cognitive training intervention. Here, we analyzed pretest performance only from tasks measuring *Gf* and experimental tasks that included experimental conditions with low and high executive processing demands (i.e., inhibition, shifting, and removal). Note that the original study additionally included tasks assessing updating, binding, and visual search. However, we did not consider these tasks here because they did not isolate specific executive processes by experimental conditions and, therefore, did not allow for separating individual differences related to the involved executive processes. Each construct was assessed by four tasks selected to represent either phonological or visuospatial content domains; 5 resulting in a total of 16 tasks analyzed in the present study. The content domain of each task was determined by its materials and processing demands as per the original study. For example, phonological tasks mainly used letters, words, or numbers as stimuli and required simple phonological processing, whereas visuospatial tasks used spatial locations or visual/figural stimuli and required visuospatial processing. Here, we only briefly describe the included tasks; for details, see the original publication.

Descriptive statistics for all measures are listed in Table 1 (*Gf*) and Table 2. An illustration for how the executive processes are isolated using SEMs is given in Figure 1. As distributions of mean RTs tend to be skewed, log-transformed RTs of correct responses were used for each condition of each executive processing task in the SEMs.

#### 2.2.1. Fluid Intelligence (*Gf*)

*Gf* was assessed by four reasoning tasks, tapping both inductive and deductive reasoning, as well as different content modalities (two verbal, one visual, and one figural task). Thus, *Gf* was assessed relatively broadly. All tasks were administered with a time limit as per their instructions. Three tasks were from the Kit of Factor-Referenced Cognitive Tests (Ekstrom et al. 1976). In the *Letter Sets Test* (30 items with a time limit of 14 min), participants had to identify the one letter set that did not fit the rule that related the other three letter sets. In the *Diagramming Relationships Test* (30 items with a time limit of 8 min), participants had to select the one Venn diagram out of five options that best matched the relationship between three nouns. In the *Locations Test* (28 items with a time limit of 12 min), participants had to choose the location for an *X* in a row, based on the spatial distribution of *X*s among dashes and blanks in the previous four rows. The fourth task was the short version of the *Raven’s Advanced Progressive Matrices* (12 items with a time limit of 15 min; Arthur and Day 1994), in which participants had to complete logical figural patterns by choosing 1 out of 8 options. For all tasks, items were grouped into three parcels containing 4 to 10 items each by computing the modulo 3 of the item indices. The average proportion of correctly solved items in each parcel served as performance indicator. Items that were not processed due to the time limit were scored as incorrect.

**Table 1:** Descriptive statistics for the average performance in the fluid intelligence measures.

Task	Content Domain	Parcel	${\mathrm{Min}}_{\mathrm{pC}}$	${\mathrm{Max}}_{\mathrm{pC}}$	$\mu_{\mathrm{pC}}$	$\sigma_{\mathrm{pC}}$
Letter Sets	PN: verbal(1)	1	0.20	1.00	0.77	0.18
		2	0.40	1.00	0.85	0.14
		3	0.20	1.00	0.79	0.19
Relations	PN: verbal(2)	1	0.20	1.00	0.71	0.17
		2	0.30	1.00	0.72	0.15
		3	0.39	1.00	0.81	0.14
Locations	VS: visual	1	0.00	1.00	0.53	0.21
		2	0.00	1.00	0.53	0.24
		3	0.00	1.00	0.56	0.21
RAPM	VS: spatial/figural	1	0.00	1.00	0.57	0.27
		2	0.00	1.00	0.62	0.27
		3	0.00	1.00	0.58	0.30

Note: PN = phonological; VS = visuospatial.

#### 2.2.2. Inhibition

Inhibition, i.e., the ability to suppress prepotent responses and filter distracting information, was assessed by a *Number Stroop* (Salthouse and Meinz 1995), a *Color Stroop* (Stroop 1935), a *Global-Local* (Navon 1977), and a *Simon* task (Simon et al. 1981). Each of these tasks required evaluating a single stimulus or multiple stimuli regarding a target feature (number value, ink color, local shape, or color), while ignoring an irrelevant feature (font size, color word, global shape, or location) that could be congruent, incongruent, or neutral with respect to the target feature. Incongruent trials required the inhibition of the irrelevant feature (indicated by the “+” in Figure 1a), whereas congruent trials facilitated the response (indicated by the “-” in Figure 1a). Neutral trials involved neither inhibition nor facilitation but controlled for all other cognitive processes included in the basic decision-making process (indicated by the “0” in Figure 1a). Thus, the variance specific to incongruent trials captures individual differences in the ability to inhibit irrelevant information.

**Table 2:** Descriptive statistics of the mean response times (in ms) for correct responses in the executive processing tasks.

Exec. Process	Task	Content Domain	Exp. Condition	${\mathrm{Min}}_{\mathrm{RT}}$	${\mathrm{Max}}_{\mathrm{RT}}$	$\mu_{\mathrm{RT}}$	$\sigma_{\mathrm{RT}}$
Inhibtion	Number Stroop	PN: numeric	congruent	403.1	898.2	579.0	106.8
			neutral	431.0	975.0	629.0	121.2
			incongruent	438.8	1032.2	669.9	116.7
	Color Stroop	PN: verbal	congruent	390.5	733.1	526.5	70.8
			neutral	398.0	746.2	542.0	72.9
			incongruent	395.1	927.9	581.1	106.4
	Global-Local	VS: visual	congruent	441.4	839.0	582.2	78.5
			neutral	447.4	903.6	600.4	85.5
			incongruent	444.1	916.9	630.9	91.4
	Simon	VS: spatial	congruent	882.0	1190.7	1001.7	62.1
			neutral	888.1	1168.3	1009.4	56.7
			incongruent	916.9	1260.3	1040.9	62.3
Shifting	Pairity-Magnitude	PN: numeric	single	419.1	761.8	556.9	64.2
			repetition	507.7	1434.1	783.5	195.6
			switch	643.0	1793.2	1107.8	221.5
	Animacy-Size	PN: verbal	single	457.0	823.7	599.2	68.2
			repetition	554.8	1507.4	904.2	217.8
			switch	772.9	1960.4	1285.9	226.5
	Color-Shape	VS: visual	single	389.6	691.1	507.4	60.0
			repetition	529.9	1589.4	935.2	225.1
			switch	787.7	1951.4	1264.5	236.3
	Fill-Frame	VS: spatial	single	390.3	665.3	502.0	56.4
			repetition	551.9	1719.2	998.9	232
			switch	791.9	2078.8	1336.9	270.6
Removal Efficiency	Digit	PN: numeric	short	429.5	2466.8	1148.1	422.1
			long	388.0	1953.3	899.9	305.7
	Letter	PN: verbal	short	459.2	3848.9	1577.6	737.6
			long	383.3	3042.8	1182.8	531.4
	Arrows	VS: visual	short	582.9	5707.2	2597.7	1110.6
			long	637.1	5407.3	2376.1	1046.0
	Locations	VS: spatial	short	1049.1	6004.0	2814.0	1059.7
			long	1071.7	5901.0	2675.1	980.1

Note: PN = phonological; VS = visuospatial.

#### 2.2.3. Shifting

Shifting, i.e., the ability to switch between different tasks, was assessed by *Parity-Magnitude*, *Animacy-Size*, *Color-Shape*, and *Fill-Frame* shifting tasks. Participants had to switch between two rules for categorizing the stimuli. For example, in the numerical content domain, participants had to switch between categorizing stimuli according to their parity (odd or even) and categorizing them according to their magnitude (smaller or larger than 5). Stimuli were bivalent for all task sets, i.e., stimuli could be evaluated on both stimulus dimensions relevant to the two tasks (e.g., a “6” presented would be both even and larger than 5). For each task set, participants completed single-task and mixed-task blocks presented in a sandwich design. First, participants completed the two tasks separately (e.g., categorizing digits only based on their parity or only based on their magnitude). Next, they completed a mixed-task block in which the two tasks switched randomly as indicated by a cue (e.g., switching between the parity and the magnitude task). Here, the task could either be the same as in the preceding trial (repetition trials), or switch to the respective other task (switch trials). Finally, participants completed the two tasks separately again but in reverse order (e.g., the magnitude task followed by the parity task).

This task setup allowed for isolating two shifting measures: (a) mixing costs, i.e., the reduction in processing speed due to the combination of two tasks, and (b) switching costs, i.e., the reduction in processing speed when switching tasks in comparison to repeating the same task in mixed blocks. As we isolated variance specific to these conditions via bi-factor models (see Figure 1b), we technically did not compute difference scores but isolated individual differences unique to blocks mixing two tasks for mixing costs and individual differences unique to trials switching between tasks in mixed blocks for shifting costs. Descriptive statistics for RTs in milliseconds in all four different tasks are shown in Table 2. Like for inhibition measures, we used log-transformed RTs for the single-task blocks and the two conditions in the mixed block (task repetition versus switch) to reduce skewness. The variance specific to the mixed-task block captures individual differences associated with the mixing of two tasks, and the variance specific to switch trials in the mixed block captures individual differences associated with the ability to switch between tasks.

#### 2.2.4. Removal Efficiency

Removal efficiency, i.e., the ability to efficiently remove information from working memory, was assessed with four tasks adapted from Ecker et al. (2014). These tasks required the initial encoding of three items (digits, letters, arrows, and colored circles) bound to a spatial location (boxes or a grid on the screen) in working memory. The initial items were then updated 1 to 16 times, one at a time. For each updating step, a cue presented first indicated which position or item was going to be updated, followed by the presentation of the new item. Participants were instructed to press the space bar as soon as they were finished with updating. The cue allowed for removing the old item before encoding the new item; critically, though, the interval between the cue and the onset of the new item (the cue-target interval; CTI) was either short (200 ms) or long (1500 ms), thereby providing either little (indicated by the “+” in Figure 1c) or plenty of time for removal (indicated by the “-” in Figure 1c) prior to the encoding of the new information.

The difference in RTs for completing the updating process (i.e., the time participants took to press the space bar after the new item was presented) for updating in steps with short and long CTIs is assumed to capture the ability to efficiently remove information from memory (Ecker et al. 2014; Singh et al. 2018). In contrast, the average time taken to update information in memory across both CTI conditions captures the general speed of updating, which includes the encoding of the new item. To ensure that participants correctly updated the information, memory was tested at the end of each trial.

### 2.3. Analysis

Data preprocessing and analysis were performed in R (R Core Team 2018) using RStudio (RStudio Team 2015). Raw data and all scripts for data preprocessing and analyses are available online at: https://osf.io/n5uv7.

#### 2.3.1. Data Preprocessing

Prior to data aggregation of mean performance and subsequent data analyses, we discarded intra- and interindividual outliers from the raw data. Specifically, for all RT-based measures (i.e., all executive processing tasks), we identified and discarded intra-individual outliers exceeding 3 median-absolute deviations from the respective individual’s median RTs. Data from participants with performance below guessing probability in any of the experimental conditions were discarded. Finally, we additionally identified and discarded univariate inter-individual outliers in each task with |z|>3. In total, this procedure resulted in discarding 1.58% of the data.

#### 2.3.2. Structural Equation Models (SEMs)

We analyzed data in two steps. First, we established measurement models for each of the four constructs: *Gf*, inhibition, shifting, and removal. These constructs isolate domain-general variance across all tasks and conditions from condition-specific and task-specific variance. Second, we merged all measurement models and estimated the relationships between the latent domain-general factors.

**Figure 2:**
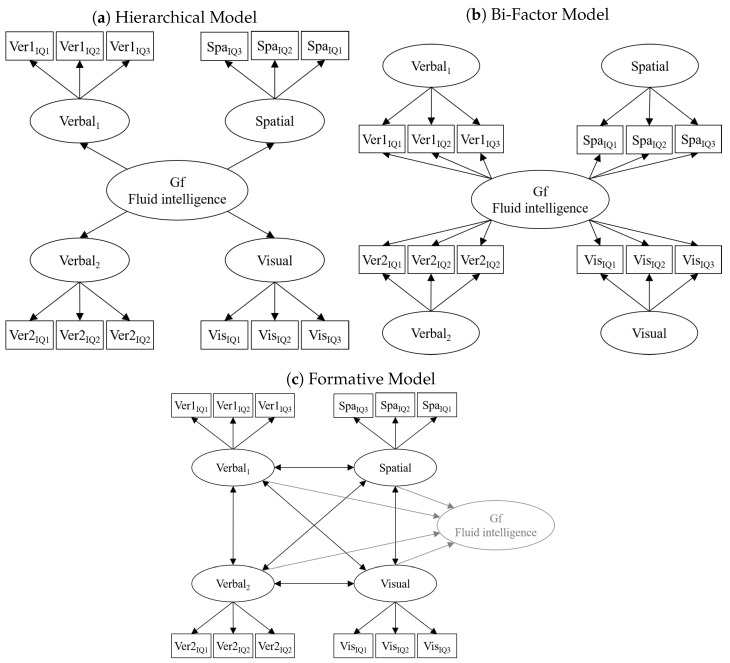
Simplified path diagrams of *Gf* models either assuming a reflective higher-order factor of *Gf* (**a**,**b**) or a formative factor *Gf* arising from correlated indicators (**c**). The two models at the top are alternative implementations of a reflective measurement model. Specifically, the hierarchical factor model assumes different levels of generality, whereas the bi-factor model reflects the breadth of a *Gf* factor. From a statistical perspective, these two models are highly similar, and the hierarchical model can be described as a special case of the bi-factor model (Gignac 2016). The bottom model assumes that factors capturing individual differences in *Gf* measures are correlated with each other, but, instead of a higher-order factor determining these correlations, a *Gf* factor emerges from these correlations. However, this emerging factor cannot be modeled in this specification because it is not identified. Error variances and residual variances of endogenous latent variables were omitted for illustration purposes.

For the measurement models of *Gf*, we evaluated which model specification, i.e., a hierarchical, a bi-factor, or a correlated factor model (see Figure 2), fit the *Gf* measures best. Different from the assumption of POT that *Gf* is a formative factor, *Gf* is implemented as a reflective factor determining the correlation between the different *Gf* factors in the hierarchical and the bi-factor model. In contrast, the correlated factor model assumes no higher-order factor as the common cause for the inter-correlations.^6^ Ideally, these three models would be compared to a fourth model that specifies a formative *Gf* factor; however, this was not possible as this model is not identified without an additional variable that is predicted by or predicts individual differences in the formative *Gf* factor (Kline 2016).

For the measurement models of executive processing tasks, we specified bi-factor models (see Figure 1). Specifically, the bi-factor models for executive processing tasks isolate domain-general variance unrelated to executive processes from domain-general variance specific to experimental conditions that more strongly demand the respective executive process. In addition, we separated task-specific variance that is shared between conditions within a given task. To identify these models, variances of the latent factors were standardized (i.e., fixed to 1), and all loadings on manifest indicators were estimated freely. To reduce the number of to-be-estimated parameters, we step-wise imposed theoretically reasonable constraints (e.g., equal error variances or loadings on factors for one measure). In sum, these models estimate the contribution of different factors to manifest performance. According to POT, they should show specific relationships between executive processing tasks and *Gf* measures.

In the second analysis step, we estimated the relationship between the latent factors from the measurement models of the executive processing tasks with the factors extracted from the *Gf* measures. We were particularly interested in the correlations between domain-general executive processing and *Gf* factors. According to POT, domain-general factors isolating executive processing variance should be most strongly related to the domain-general *Gf* factor. Although we estimated correlations between task-specific factors, this was not the primary focus of the current study because POT assumes that *Gf* primarily samples domain-general executive processes.

For all SEMs, the observed variables were standardized prior to model estimation. Although this is not always recommended, we did so to avoid an ill-defined covariance matrix due to highly diverging variances between RT and accuracy measures included in the models. SEMs were estimated with a full-information maximum likelihood estimator. Model fit was evaluated using the $\chi^{2}$-statistic, as well as CFI and RMSEA as fit indices, as in Hu and Bentler (1998, 1999).

## 3. Results

### 3.1. Experimental Effects in the Executive Processing Tasks

First, we examined whether the executive processing tasks indeed tapped the theoretically assumed processes. For this purpose, we ran ANOVAs testing on whether the experimental manipulations yielded the expected effects using the *afex* packages in R (Singmann et al. 2018).

#### 3.1.1. Inhibition Effects

For all four inhibition tasks, RTs differed between the congruency conditions, $F\left(2,\;434\right)=1047.2,\;p<.001,\;\epsilon_{GG}=.84,\;\omega^{2}=.11,\;95\%$ CI =[.06, .16]. RTs also differed between tasks, $F\left(3,\;651\right)=3175.7,\;p<.001,\;\epsilon_{GG}=.75,\;\omega^{2}=.83,\;95\%$ CI =[.77, .85], as did the difference between congruency conditions, $F\left(6,\;1302\right)=57.6,\;p<.001,\;\epsilon_{GG}=.75,\;\omega^{2}=.01,\;95\%$ CI =[.00, .03]. Specifically, whereas the inhibition effect (i.e., the difference in RT between neutral and incongruent conditions) was numerically similar across the four tasks (28.6 ms to 40.9 ms), the facilitation effect (i.e., difference in RT between neutral and congruent conditions) was largest for the numerical inhibition task (49.7 ms) and considerably smaller for the other three inhibition tasks (9.6 ms to 18.9 ms). Nonetheless, differences between congruency conditions were statistically significant in all four tasks (see Supplementary Online Material: https://osf.io/n5uv7).

#### 3.1.2. Shifting Effects

In the shifting tasks, RTs differed between the single-task trials and mixed-tasks trials repeating the same task, $F\left(1,\;223\right)=1349.7,\;p<.001,\;\omega^{2}=.65,\;95\%$ CI =[.59, .71]. This effect reflects so-called mixing costs that arise due to combining two tasks rather than processing only a single task. The magnitude of mixing costs differed between shifting tasks, $F\left(3,\;669\right)=210.2,\;p<.001,\;\epsilon_{GG}=.90,\;\omega^{2}=.16,\;95\%$ CI =[.11, .21]. Specifically, mixing costs were smaller in the numerical and verbal tasks (220 and 295 ms) than in the visual and spatial tasks (418 and 490 ms). Still, mixing costs were statistically significant for each task (see Supplementary Online Material: https://osf.io/n5uv7). In addition, RTs in mixed-task trials differed between task repetitions and task switches, reflecting switching costs, $F\left(1,\;215\right)=1396.6,\;p<.001,\;\omega^{2}=.47,\;95\%$ CI =[.38, .55]. The size of switching costs also differed significantly between shifting tasks, $F\left(3,\;645\right)=8.9,\;p<.001,\;\epsilon_{GG}=.98,\;\omega^{2}=.00,\;95\%$ CI =[.00, .01], but these differences were negligible on an absolute level (322 to 381 ms). Likewise, all switch cost were statistically significant for all tasks individually (see Supplementary Online Material: https://osf.io/n5uv7).

#### 3.1.3. Removal Efficiency Effects

In the removal tasks, participants needed longer to remove old and encode new information into working memory when the CTI was short than when it was long, $F\left(1,\;207\right)=488.9,\;p<.001,\;\omega^{2}=.05,\;95\%$ CI =[.01, .11]. Again, RTs differed between tasks, $F\left(3,\;621\right)=313.5,\;p<.001,\;\epsilon_{GG}=.75,\;\omega^{2}=.46,\;95\%$ CI =[.41, .51], as did the difference between short and long CTIs, $F\left(3,\;621\right)=39.1,\;p<.001,\;\epsilon_{GG}=.85,\;\omega^{2}=.00,\;95\%$ CI =[.00, .02]. Specifically, removal efficiency was highest (i.e., the difference between short and long CTI conditions was smallest) for the spatial task (119 ms). For the visual and numerical task, removal efficiency was lower (194 ms and 235 ms), and it was lowest for the verbal task (356 ms). Nonetheless, removal costs were statistically significant within each individual task (see Supplementary Online Material: https://osf.io/n5uv7).

Taken together, the typical patterns of experimental effects reflecting the three executive processes were observed in all tasks, indicating that the experimental manipulations tapped the executive processes targeted. The experimental effects were largest for the shifting measures and relatively smaller for the inhibition and removal efficiency measures. The effect size may affect the amount of variability in experimental effects that can be isolated in the executive processing tasks (Rouder and Haaf 2019). More details on the analysis, particularly the post hoc tests, and descriptive plots are provided in the analysis script in the Supplementary Materials on OSF (https://osf.io/n5uv7).

### 3.2. Measurement Models

Next, to assess the extent to which *Gf* and executive processing measures captured individual differences in domain-general and domain-specific processes, we examined the measurement models for each construct using the bi-factor models introduced in the *Analysis* section. These models, as well as all further structural equation models, were estimated using the *lavaan* package in R (Rosseel et al. 2018).

#### 3.2.1. Fluid Intelligence (*Gf*)

We compared the three measurement models for the *Gf* measures described in the method section (see Figure 2):

1. a hierarchical measurement model assuming a general *Gf* factor,
2. a bi-factor measurement model separating a general *Gf* factor from task-specific factors, and
3. a measurement model assuming correlated first-order factors not specifying a higher-order factor of *Gf*.

In addition, we simplified all three models in a step-wise procedure. First, we assumed tau-equivalent measurement models with equal error variances for the four *Gf* measures (constraint I). Second, we assumed tau-equivalence measurement on the higher order level, meaning that all four intelligence measures equally measured *Gf* (constraint II). This second assumption was implemented by setting either the general *Gf* factor to load equally on all indicators or lower-level factors or, for the correlated-factors model, the correlations between all lower-level factors to be equal.

The model fit for all measurement models is summarized in Table 3. In general, the unconstrained, as well as the two constrained, specifications of the three different models fitted the data well. Overall, the hierarchical model was more parsimonious than the other two models, while still fitting well to the data. Including the additional constraints in the three models did not significantly deteriorate model fit for any of them (see likelihood ratio tests, and AIC and BIC in Table 3). Moreover, including the addition constraints reduced the differences in fit between the three measurement models. In particular, for the most restrictive models assuming tau-equivalence for each *Gf* measure and equal factor loadings or factor correlations, the fit was equal for the hierarchical and the correlated-factor model, and only slightly worse for the bi-factor model.

From an empirical perspective, this indicates that, despite assuming different theoretical models of *Gf*, statistically, these models can hardly be distinguished in cross-sectional data. Furthermore, given the almost equal fit of all three models, the hierarchical model and the bi-factor model should capture the same shared variance among the *Gf* measures as the correlated-factor model does. Although technically neither of these two models assumes a formative *Gf* factor, from our perspective, all three models can serve the purpose of testing whether different executive processes independently predict individual differences in *Gf*. To keep the measurement model of *Gf* comparable to the measurement models of the executive processing tasks, we used the bi-factor model in the following analyses.

In the maximally constrained bi-factor model for *Gf* measures, the latent factors explained between 41 to 49% of variance in the manifest indicators, suggesting a large portion of variance in each indicator not explained by the latent factors. This could be due to either (a) specific processes that are not shared with the other indicators or (b) measurement error due to the relatively low number of trials for each indicator. Nonetheless, the reliability of the general *Gf* factors was acceptable (ω=.85, estimated as proposed by Bollen (1980) and Raykov (2001)). In contrast, reliability of the task-specific was considerably lower (ω = .45–.58). However, the proportion of explained variance in manifest indicators is comparable to other factor models separating domain-general and task-specific variances in intelligence measures (Johnson et al. 2008). Specifically, the loading on the general *Gf* factor of the indicators (β=.51) was comparable to loadings on the task-specific factors (β = .40–.48). Likewise, error variances were comparable across the four *Gf* measures (ϵ = .51–.59). For more details regarding the model comparisons and results, see the online supplement.

#### 3.2.2. Inhibition

The unconstrained bi-factor model separating domain-general and task-specific variance for the inhibition tasks (see Figure 1a) accounted well for the data, $\chi^{2}\left(46\right)=41.4,p=.665$, CFI=1.000, RMSEA=.000 (95%CI=[.000, .036]). However, factor reliabilities were low for the factors isolating individual differences in domain-general executive processing ($\omega_{\mathrm{Inhib}.}=.20$; $\omega_{\mathrm{Fac}.}=.16$). This is also reflected in loadings on these factors not differing significantly from zero (see Table 4). For the domain-general speed factor, as well as all task-specific factors, reliabilities were good (ω = .94–.99).

To address these problems, we fixed non-significant loadings and two non-significant error variances to zero and refit the model. Although this second model fit the data worse, $\Delta \chi^{2}\left(10\right)=22.97,\;p=.011$, the other fit indices still suggested an acceptable fit to the data, CFI=.998, RMSEA=.025 (95%CI=[.000, .049]). Next, we evaluated how imposing additional, theoretically reasonable, constraints would affect model fit. A third model assuming a tau-equivalent measurement of the domain-general speed factor, and verbal and spatial task-specific factors did not further deteriorate model fit, $\Delta \chi^{2}\left(15\right)=13.7,\;p=.547$, and still fit acceptably to the data, $\chi^{2}\left(71\right)=78.1,\;p=.264$, CFI=.999, RMSEA=.021 (95%CI=[.000, .044]), Thus, this third model was retained for further analyses.

In this third model, the latent factors explained between 87 to 100% of the variance in the indicators. The domain-general speed factor captured the greater proportion of this variance (50–65%) than task-specific factors (35–50%). In contrast, loadings of neither the inhibition nor the facilitation factor on the indicators were statistically significant, suggesting that there is little domain-general variance that can be attributed to specific executive processes in inhibition tasks.

#### 3.2.3. Shifting

The unconstrained bi-factor model isolating variance proportions for domain-general and task-specific processes in the shifting tasks (see Figure 1b) fit the data acceptably, $\chi^{2}\left(42\right)=53.80,\;p=.105$, CFI=.995, RMSEA=.034 (95%CI=[.000, .059]). Factor reliabilities were acceptable for domain-general ($\omega_{Spd}=.97$; $\omega_{Mix}=.91$, and $\omega_{Shift}=.80$) and slightly lower, but still acceptable, for the task-specific factors (ω = .68–.77). Nevertheless, a few loadings and error variances did not differ significantly from zero (see Table 4). In a second model, these were fixed to zero, and the model was refit. Model fit for this second model did not differ significantly from the initial model, $\Delta \chi^{2}\left(3\right)=3.41,\;p=.333$, CFI=.995, RMSEA=.034 (95%CI=[.000, .058]). We were unable to impose additional theoretically reasonable constraints to this model without considerably deteriorating model fit. Thus, this second model was retained for further analyses.

The latent factors in this second model explained 71 to 100% of variance in the manifest indicators. Specifically, the domain-general speed factor explained 65 to 85% of variance in RTs from single-task blocks and 30 to 45% of variance in RTs from mixed-task blocks. Variance in RTs from mixed-task blocks was additionally explained by the domain-general mixing factor (25–35% for task repetitions, 5–10% for task switches) and the domain-general shifting factor (10–25%). The task-specific factors captured the smallest proportion of variance in RTs (between 2–25%).

#### 3.2.4. Removal Efficiency

The bi-factor model isolating domain-general speed and removal efficiency from task-specific processes in the removal tasks (see Figure 1c) accounted well for the data, $\chi^{2}\left(20\right)=27.98,\;p=.110$, CFI=.997, RMSEA=.041 (95%CI=[.000, .074]). Factor reliabilities were acceptable for the domain-general factors ($\omega_{Spd}=.99$; $\omega_{Rem}=.72$), and for all task-specific factors ($\omega_{s}=.95-.99$), except for the numerical factor (ω=.00). This was also reflected in loadings on the numerical factor being zero. As for the previous model, we fixed these loadings, as well as other non-significant loadings or error variances, to zero and refit the model. Fixing the loading and error variances to zero did not worsen the fit for this second model, $\Delta \chi^{2}\left(5\right)=3.24,\;p=.663$, CFI=.997, RMSEA=.032 (95%CI=[.000, .064]). Additionally, we fixed the loadings for the numerical, visual, and spatial indicators on the domain-general speed factor to be equal within each domain, assuming that both conditions within each domain was equally determined by the domain-general speed factor. This additional constraint did not deteriorate model fit, $\Delta \chi^{2}\left(3\right)=3.31,\;p=.346$, CFI=.997, RMSEA=.031 (95%CI=[.000, .062]); thus, we retained this third model for further analyses.

The latent factors of this third bi-factor model for removal efficiency captured 90 to 100% of the variance in manifest indicators. Specifically, the domain-general speed factor captured 10 to 100% of variance. The domain-general removal factor captured 5 to 10% of variance, but only for numerical and verbal tasks. The loadings of the removal factor on visual conditions was significant but negligible, and, on spatial conditions with short CTI, it was not significantly different from zero, suggesting that they did not share any domain-general variance specific to the short CTI condition with the numerical and verbal removal task. In contrast, the task-specific factors captured 60 to 90% of variance, suggesting that there is a large proportion of task-specific variance in RTs associated with removal.

### 3.3. Relationships between Performance in Executive Processing Tasks and Intelligence Measures

The joint SEM for the 12 executive processing tasks tapping three different executive processing aspects (i.e., inhibition, shifting, and removal efficiency) fitted acceptably to the data, $\chi^{2}\left(466\right)=675.46,\;p<.001$, CFI=.980, RMSEA=.044 (95%CI=[.036, .051]). The model results suggested that there is strong overlap between general processing speed in inhibition and shifting tasks (r=1.00); 7 whereas the relationship of general processing speed in both inhibition and shifting tasks with processing speed in the removal tasks was considerably smaller (r=.39).^8^ Furthermore, relationships between domain-general factors of executive processes (i.e., mixing, shifting, and removal efficiency) were not significantly different from zero, all $p_{s}>.489$. Finally, there were only small and inconsistent relationships between the task-specific factors (all $r_{s}<.21$), except for the relationship between the spatial and visual removal factor, r=.33, and the numerical inhibition with the numerical shifting factor, r=.25. In a second model, we constrained all parameters that were not significantly different from zero to zero and refit the model. This second model did not fit the data worse than the unconstrained model, $\Delta \chi^{2}\left(10\right)=15.58,\;p=.112$, CFI=.979, RMSEA=.044 (95%CI=[.036, .051]).

**Figure 3:**
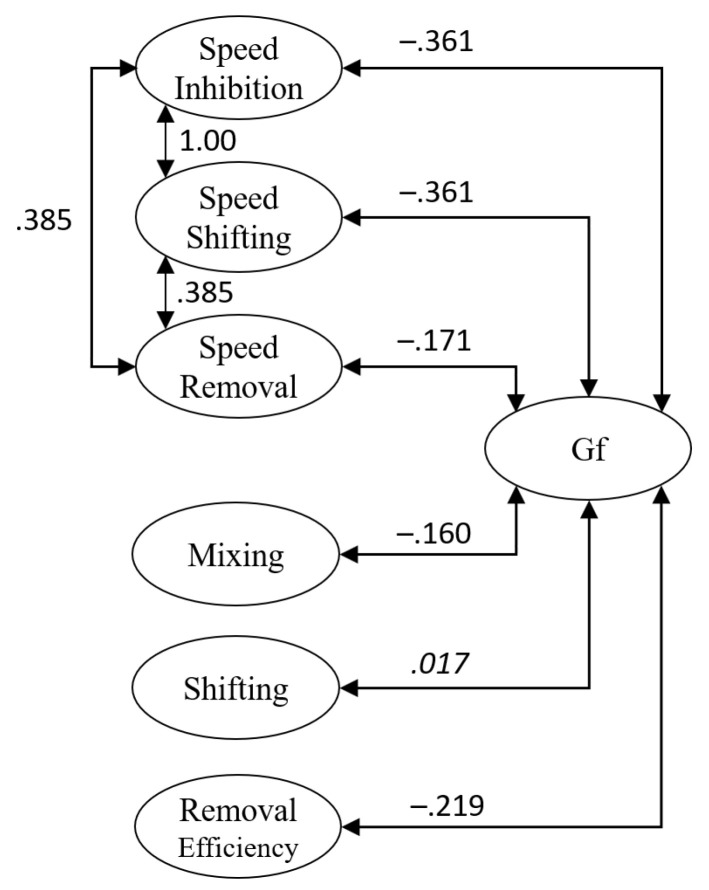
Illustrations of the relationships between domain-general factors from the executive processing tasks with the domain-general *Gf* factor extracted from *Gf* measures. Parameters in *italics* did not differ significantly from zero.

We then merged this joint model of the executive processing tasks with the bi-factor measurement model for the *Gf* measures. We estimated all correlations between domain-general factors extracted from the executive processing tasks with the *Gf* factors, as well as correlations between the task-specific factors of the phonological and the visuospatial content domain. This joint model of performance in the executive processing tasks and *Gf* measures showed an acceptable fit to the data, $\chi^{2}\left(914\right)=1205.02,\;p<.001$, CFI=.974, RMSEA=.037 (95%CI=[.031, .042]). The correlations between domain-general factors from the executive processing tasks with the *Gf* factor are displayed in Figure 3. As the general processing speed factors derived from inhibition and shifting measures were correlated perfectly (r=1.00), we constrained the correlations of these two factors with the general *Gf* factor to be equal. The speed factors from inhibition and shifting tasks were negatively correlated (r=−.36) with *Gf*, i.e., people showing faster speed showed better performance in the *Gf* measures. This correlation was smaller but still significant for the relationship of processing speed in removal tasks and the *Gf* factor (r=−.17). For the domain-general factors isolating variance specific to executive processes, the relationships were smaller. Specifically, the correlation between shifting and *Gf* was not significantly different from zero, with p=.839, and the correlation between mixing and *Gf* was significant but small (r=−.16), as was the correlation between removal efficiency and *Gf* (r=−.22). Thus, on the domain-general level, *Gf* was most strongly related to processing speed in inhibition and shifting tasks and, although to a lesser extent, to speed in removal tasks, and variance specific to the executive processes involved in mixing and removal.

With respect to the task-specific factors, there was only one significant correlation between variance specific to the visual removal task with differences in the figural *Gf* measure, r=.278. All other correlations were not significantly different from zero, indicating that there were little consistent relationships on a task-specific level that could be attributed to materials from similar content domains. In fact, setting all non-significant correlations to zero did not worsen model fit, $\Delta \chi^{2}\left(22\right)=15.03,\;p=.861$, CFI=.974, RMSEA=.036 (95%CI=[.030,.041]). Taken together, there was little overlap between task-specific factors from the executive processing tasks with task-specific aspects of the intelligence measures.

## 4. Discussion

POT proposes that *Gf* measures sample executive processes. The aim of the present study was to identify these executive processes. For this purpose, we isolated domain-general factors for processing speed and executive processing from executive processing tasks, and assessed to what extent these factors are related to the shared variance from *Gf* measures. According to POT, *Gf* is thought to primarily represent the shared domain-general executive processes sampled by *Gf* measures. Thus, the domain-general *Gf* factor should be related to the factors isolating variance specific to experimental manipulations that specifically required executive processes. Our results did not confirm these expectations. Instead, domain-general processing speed, but not domain-general executive processing, showed the largest correlation with *Gf*, and the relationships of executive processing factors with *Gf* were smaller and inconsistent.

### 4.1. Isolating Variance from Domain-General Cognitive Processes in Executive Processing Tasks

Our results suggest that domain-general executive processing factors isolating variance specific to experimental conditions that strongly require executive processing contribute only little variance to overall performance in inhibition and removal tasks. Instead, domain-general processing seems to contribute more strongly to individual differences in these tasks. This is in line with a growing number of studies indicating that there are little individual differences in the experimental effects of inhibition tasks (Frischkorn et al. 2019; Rey-Mermet et al. 2018, 2019; Stahl et al. 2014). Still, other researchers argue that these results rather indicate that tasks measuring inhibition by reaction time differences are not ideally suited to capture individual differences in inhibition reliably (Draheim et al. 2021). For efficiency of removal from working memory, we were only able to isolate variance specific to removal efficiency for verbal, numerical, and—to a lesser extent—visual tasks. Notably, previous studies reporting individual differences in removal efficiency also administered only verbal and numerical tasks (Rey-Mermet et al. 2020; Singh et al. 2018). In contrast, the factors isolating processes associated with mixing (i.e., performing two tasks at once) and processes specific to switching (i.e., alternating between two tasks) contributed to a similar degree to overall performance in shifting tasks as the domain-general speed factor. This confirms that measuring individual differences in mixing and switching, as well as removal abilities for verbal tasks, is not as problematic as measuring inhibition or removal abilities for visual or spatial tasks (von Bastian et al. 2020; von Bastian and Druey 2017).

Across all executive processing tasks, task-specific factors captured a considerable proportion of variance in manifest performance. Specifically, task-specific factors captured the relatively largest proportion of variance in the removal tasks, followed by the inhibition tasks and then the shifting tasks. Although materials for the tasks tapping the three different executive processes were selected to tap similar content domains, the small and inconsistent correlations between task-specific factors tapping similar content domains suggest that these factors captured mostly task but not domain-specific, individual differences. Nonetheless, these results suggest that task-specific cognitive processes contribute considerably to performance in executive processing tasks.

Taken together, our results are in line with numerous previous studies demonstrating difficulties in isolating variance specific to executive processing, particularly in inhibition (Frischkorn et al. 2019; Hedge et al. 2018; Rey-Mermet et al. 2018, 2019; Stahl et al. 2014; von Bastian et al. 2016). Some researchers have argued that this problem is mainly due to the use of difference measures computed by contrasting conditions with high executive processing demands and conditions with low executive processing demands (Draheim et al. 2019). Difference measures tend to show lower reliability (Cronbach and Furby 1970; Hedge et al. 2018) when performance across conditions is highly correlated. Still, even in studies where difference measures showed acceptable reliability, or where latent variable models were used to isolate only reliable differences between conditions, the reported correlations among executive processing measures were only weak (Frischkorn et al. 2019; Rey-Mermet and Gade 2018). In the present study, we addressed the problem of low reliability by using SEMs that extract only reliable variance specific to those experimental conditions with high executive processing demands. Yet, factor reliabilities for executive processing factors tended to be low, particularly for the executive processing factors in the inhibition tasks. Thus, although we were unable to overcome all problems of measuring executive processes (Draheim et al. 2019; Rouder and Haaf 2019), we still avoided the possible attenuation of relationships of individual differences in executive processes due to low reliability by extracting only reliable individual differences specific to executive processing manipulations.

Those executive processes that we were able to reliably measure—mixing, switching, and efficiency of removal of information from WM—were unrelated to each other. This is in line with POT’s assumption that the different executive processes underlying individual differences in *Gf* are independent. However, at first glance, it seems to contradict the common executive processes model (Friedman and Miyake 2017; Friedman et al. 2016; Karr et al. 2018; Miyake et al. 2000) that found considerable overlap of inhibition with shifting and updating. Obviously, as we were unable to extract reliable individual differences in inhibition, it was impossible to detect overlap of inhibition with shifting and updating. Still, the domain-general executive processing factors we extracted are more comparable with the shifting-specific and updating-specific factors that Miyake, Friedman, and colleagues extracted, and they were also unable to establish an inhibition-specific factor. Moreover, we found that the domain-general factors reflecting general processing speed in inhibition and shifting tasks were indistinguishable. As the modeling approach taken in the common executive processes model by Miyake, Friedman, and colleagues does not account for domain-general individual differences in processing speed, it is unclear at this point to what extent the common executive processing factor in their model may also largely reflect general processing speed rather than domain-general executive processes.

Our finding that domain-general factors tapping general processing speed were strongly correlated for inhibition and shifting tasks suggests that inhibition and shifting either sample the same set of independent cognitive processes underlying general processing speed, or that they rely on the same cognitive processing speed that causally determines individual differences in inhibition and shifting tasks. As RTs are determined by multiple factors, domain-general processing speed might also reflect individual differences in multiple cognitive processes. The present analyses are not suitable for investigating whether the domain-general processing factors extracted from inhibition and shifting tasks reflect a single process or a set of multiple independent cognitive processes. Nevertheless, other studies investigating domain-general and domain specific aspects of cognitive processing speed do suggest that there is a large overlap in the speed of evidence accumulation across different speeded choice tasks (Lerche et al. 2020).

In summary, using latent bi-factor models enabled us to isolate reliable individual differences in the executive processes shifting and removal from working memory, but not inhibition. Shifting and removal were largely uncorrelated with all other factors specific to executive processes, which supports the assumption of POT that executive processes are independent. However, only the variance that reflected cognitive processes associated with general processing speed, but not the variance specific to experimental manipulations aimed to engage executive processes, was highly correlated for inhibition and shifting tasks.

### 4.2. Relationships of Domain-General Processing Speed and Executive Processes with *Gf* Measures

Our results suggest that mainly the domain-general factors isolating general processing speed in the executive processing tasks were moderately associated with the domain-general variance shared across different *Gf* measures (r=−.17to−.36). Domain-general factors isolating variance specific to executive processes showed smaller correlations with *Gf* (r=.01to−.22). Hence, while the independence of the domain-general factors isolating variance for executive processes is in line with POT, their lack of overlap with *Gf* is not. In contrast, the statistically indistinguishable domain-general processing speed factors from the inhibition and shifting measures, did overlap with *Gf* to some extent, suggesting that they are also sampled by *Gf* measures. It is important to note, however, that the *Gf* measures were administered with a time limit. Given that time restrictions might increase the contribution of processing speed to individual differences in the *Gf* measures, this correlation could be overestimated. Nevertheless, there is considerable evidence indicating moderate correlations of processing speed with *Gf* (e.g., Schmiedek et al. 2007; Sheppard and Vernon 2008), which is in line with the relationship we found in this study. Therefore, our findings provide evidence that *Gf* measures sample general processing speed rather than executive processes. Although this finding can be reconciled with POT, it does contradict theoretical perspectives assuming that executive processes, such as the disengagement from irrelevant information, are critical for individual differences in *Gf* (Shipstead et al. 2016).

Furthermore, we found that there is little overlap between the task-specific factors isolated in executive processing tasks and *Gf* measures. If these factors captured some domain-specific processes, this finding would contradict POT’s assumption of some overlap in the processes required by tasks and measures tapping the same content domain. However, as mentioned earlier, because we administered only 1 to 2 tasks for each content domain, it is possible that these task-specific factors did primarily capture task-specific instead of content domain-specific cognitive processing. Thus, these factors could also represent the unique cognitive processes that are not sampled by other tasks in the current study. There would likely be only little overlap between task-specific cognitive processing and *Gf* measures, thus explaining why the correlations were small. Nonetheless, even if task-specific differences may have attenuated the correlations between domain-specific factors from executive processing tasks and intelligence measures, we still would have expected to obtain consistently small yet significant correlations among them.

One limitation of the current study is that our measurement of executive processes solely relied on reaction times. Although empirical results indicated that processing speed is correlated with intelligence differences (Sheppard and Vernon 2008), performance in intelligence tests does mainly require correctly solving test items, not primarily how quickly an individual does so. Thus, using executive processing measures that focus on accuracy might increase the relationship of executive processes with intelligence (Draheim et al. 2019, 2021). Nevertheless, there is still considerable discussion on how to best measure executive processes (von Bastian et al. 2020). Therefore, it is not clear whether a simple shift from reaction time-based measurement to accuracy-based measurement actually solves this problem. For example, a recent study that isolated updating specific processes with accuracy-based measures still found little overlap of updating specific processes with intelligence or working memory capacity (Frischkorn et al. 2020).

## 5. Conclusions

POT proposes that *Gf* primarily represents the shared executive processes sampled by different *Gf* measures (Kovacs and Conway 2016). In the present study, we separated individual differences specific to experimental manipulations requiring executive processes. Our results showed that factors isolating domain-general executive processes were only moderately related to individual differences in *Gf*. Instead, we found the largest overlap between *Gf* measures with domain-general processing speed factors, particularly processing speed in inhibition and shifting tasks. This seems to contradict POT as the domain-general processing speed variance from inhibition and shifting tasks were not independently related to *Gf* differences. Yet, to align these results with POT, one could still argue that the shared general speed variance of inhibition and shifting tasks possibly reflects multiple independent cognitive processes. These same cognitive processes could then, to a lower extent, be sampled by *Gf* measures, thereby explaining the moderate negative correlation between the general speed factor and the domain-general *Gf* factor. However, this line of argumentation, namely that strong overlap between factors from basic cognitive processing tasks reflects their impurity in measuring a single cognitive processes, could be applied to almost any study showing strong correlations between performance factors extracted from cognitive processing tasks. This demonstrates the difficulty in actually testing POT as long as there is no adequate and agreed upon method for isolating single cognitive processes.

Ultimately, therefore, these results highlight both problems in the measurement of executive processes, as well as the considerable room for interpretation in POT. As long as we lack theoretically grounded methods to measure individual differences in specific executive processes, strong correlations between different executive processing factors can always be reconciled assuming that we did not sufficiently isolate the cognitive processes that are sampled by the correlated factors. Therefore, to adequately test POT, we need to improve the measurement of single cognitive processes while also refining and specifying which cognitive processes are assumed to be sampled by intelligence measures according to POT.

## Figures and Tables

**Table 3:** Model fit for measurement models of fluid intelligence measures.

Model	Constraints	$\chi^{2}$	df	*p*	CFI	RMSEA	AIC	BIC	$\Delta \chi^{2}$	*p*
Hierarchical	no	47.5	62	.914	1.00	.00 [.00; .02]	7353.0	7450.1		
	I	61.2	78	.920	1.00	.00 [.00; .01]	7334.7	7376.3	13.7	.621
	I & II	61.5	81	.947	1.00	.00 [.00; .00]	7329.1	7360.3	0.4	.946
Bi-Factor	no	38.9	54	.940	1.00	.00 [.00; .01]	7360.4	7485.2		
	I	57.9	70	.848	1.00	.00 [.00; .02]	7347.5	7416.8	19.1	.264
	I & II	62.4	81	.938	1.00	.00 [.00; .00]	7329.9	7361.1	4.4	.956
Correlated	no	44.9	60	.926	1.00	.00 [.00; .01]	7354.5	7458.5		
	I	59.2	76	.922	1.00	.00 [.00; .01]	7336.8	7385.3	14.3	.577
	I & II	61.5	81	.947	1.00	.00 [.00; .00]	7329.1	7360.3	2.3	.805

**Table 4:** Factor loadings and error variances for the unconstrained measurement models of the executive processing tasks.

	**Indicator**		**Domain-General Factors**			**Task-Specific Factors**				
	**Task**	**Condition**	**Speed**	**Inhibition**	**Facilitation**	**Num**	**Ver**	**Vis**	**Spa**	**Error**
Inhibition	Numerical	neutral	.71			.73				*.00*
		congruent	.72		*.06*	.67				.04
		incongruent	.74	*.06*		.66				.05
	Verbal	neutral	.78				.61			.03
		congruent	.79		*.06*		.58			.04
		incongruent	.74	*.13*			.61			.08
	Visual	neutral	.76					.59		.10
		congruent	.78		*.11*			.63		*.02*
		incongruent	.78	*.03*				.59		.08
	Spatial	neutral	.80						.62	.03
		congruent	.78		*−.04*				.57	.08
		incongruent	.77	*.05*					.60	.08
	**Indicator**		**Domain-General Factors**			**Task-Specific Factors**				
	**Task**	**Condition**	**Speed**	**Mixing**	**Shifting**	**Num**	**Ver**	**Vis**	**Spa**	**Error**
Shifting	Numerical	single task	.86			.31				.21
		mixed repeat	.57	.48		.54				.18
		mixed switch	.58	.33	.45	.45				.17
	Verbal	single task	.92				.16			.17
		mixed repeat	.67	.61			.35			*.07*
		mixed switch	.64	.27	.47		.43			.14
	Visual	single task	.91					.20		.16
		mixed repeat	.54	.64				.46		*.08*
		mixed switch	.54	.29	.51			.36		.24
	Spatial	single task	.84						.13	.29
		mixed repeat	.57	.52					.70	*−.04*
		mixed switch	.63	.23	.36				.46	.24
	**Indicator**		**Domain-General Factors**			**Task-Specific Factors**				
	**Task**	**Condition**	**Speed**	**Removal**		**Num**	**Ver**	**Vis**	**Spa**	**Error**
Removal	Numerical	long CTI	.99			$.00{\;}^{a}$				.09
		short CTI	.98	.32		$.00{\;}^{a}$				.00 *
	Verbal	long CTI	.76				$.68{\;}^{b}$			*.01*
		short CTI	.68	.23			$.68{\;}^{b}$			.09
	Visual	long CTI	.36					$.93{\;}^{c}$		*.01*
		short CTI	.33	.05				$.93{\;}^{c}$		.06
	Spatial	long CTI	.38						$.93{\;}^{d}$	.03
		short CTI	.36	*.02*					$.93{\;}^{d}$	*.01*

Note: Loadings and error variances that were not significantly different from zero are displayed in *italic*. Num = numerical; Ver = verbal; Vis = visual; Spa = spatial. Parameters that are indexed with the same superscript (e.g., *^a^*) were constrained to be equal. * This error variance was fixed to zero in the unconstrained model because it was estimated to be negative.

## Data Availability

Raw Data and scripts for data preparation and analysis are available online: https://osf.io/n5uv7.

## Notes

1 POT does not assume the specific executive processes to be perfectly independent. In this, small correlations between the executive processing factors would not contradict POT.

2 Notably, results from other studies suggest that memory tasks only involving storage demands are equally—if not better—suited measures of individual differences in working memory capacity than complex span tasks and show similarly strong associations to intelligence measures (Wilhelm et al. 2013; Colom et al. 2005, 2008). It is, therefore, still a matter of debate to what extent executive processes limit the capacity of working memory (Oberauer et al. 2016; Shipstead et al. 2016).

3 Other common updating tasks, such as the N-back or keep-track tasks, measure updating as the accuracy of correct memory retrieval in WM tasks involving updating. In contrast, measures of removal efficiency indicate how long an individual takes to update information after having been cued to update one specific item in working memory. Therefore, the RT-based measure of removal efficiency more specifically isolates individual differences specific to the process of removing outdated information from memory (Ecker et al. 2014) and does not conflate basic WM maintenance abilities with updating specific processes (Frischkorn et al. 2020).

4 The term *disengagement* more generally refers to inhibition or removal of outdated or irrelevant information. Thus, disengagement is implemented by the three studied executive processes in different forms.

5 The original study (De Simoni and von Bastian 2018) distinguished between numerical, verbal, visual, and spatial tasks; however, in hindsight, we noted some problems with this distinction. For example, arguably, the Raven’s Advanced Progressive Matrices should be classified as a figural task, and the Locations Test could just as well be classified as a spatial instead of a visual task. Therefore, for the purpose of the present study, we decided to group verbal & numerical tasks into *phonological* tasks and visual, spatial, and figural tasks into *visuospatial* tasks.

6 Although a higher-order model could always be fit to a correlated factor model (Gignac and Kretzschmar 2017), some researchers argue that a correlated factor model more explicitly indicates that there are distinct but correlated dimensions (Van Der Maas et al. 2006). Additionally, there can be statistically equivalent models that still differ in their theoretical interpretation; thus, we cannot ground the interpretation of SEM solely on the existence of equivalent or other well-fitting models (Borsboom 2005; Borsboom et al. 2003).

7 In fact, this correlation was fixed to one because it was estimated to be larger than one.

8 As general processing speed in inhibition and shifting tasks was perfectly correlated, this correlation was constrained to be equal between general processing speed in inhibition and removal tasks, as well as in shifting and removal tasks.
